# Dynamic Network of Interactions in the Wildlife-Livestock Interface in Mediterranean Spain: An Epidemiological Point of View

**DOI:** 10.3390/pathogens9020120

**Published:** 2020-02-13

**Authors:** Roxana Triguero-Ocaña, Beatriz Martínez-López, Joaquín Vicente, José A. Barasona, Jordi Martínez-Guijosa, Pelayo Acevedo

**Affiliations:** 1 Instituto de Investigación en Recursos Cinegéticos (IREC) UCLM-CSIC-JCCM, 13071 Ciudad Real, Spain; joaquin.vicente@uclm.es (J.V.); jordi.m.guijosa@gmail.com (J.M.-G.);; 2 Center for Animal Disease Modeling and Surveillance, Department of Medicine and Epidemiology, School of Veterinary Medicine, University of California-Davis, Davis, CA 95616, USA; beamartinezlopez@ucdavis.edu; 3 Escuela Técnica Superior de Ingenieros Agrónomos, UCLM, 13071 Ciudad Real, Spain; 4 VISAVET, Animal Health Department, Complutense University of Madrid, 28040 Madrid, Spain; joseangel.barasona@gmail.com

**Keywords:** social network analysis, GPS, proximity loggers, wild ungulates, cattle, tuberculosis, interactions, epidemiology, multi-host system

## Abstract

The correct management of diseases that are transmitted between wildlife and livestock requires a reliable estimate of the pathogen transmission rate. The calculation of this parameter is a challenge for epidemiologists, since transmission can occur through multiple pathways. The social network analysis is a widely used tool in epidemiology due to its capacity to identify individuals and communities with relevant roles for pathogen transmission. In the present work, we studied the dynamic network of interactions in a complex epidemiological scenario using information from different methodologies. In 2015, nine red deer, seven fallow deer, six wild boar and nine cattle were simultaneously monitored using GPS-GSM-Proximity collars in Doñana National Park. In addition, 16 proximity loggers were set in aggregation points. Using the social network analysis, we studied the dynamic network of interactions, including direct and indirect interactions, between individuals of different species and the potential transmission of pathogens within this network. The results show a high connection between species through indirect interactions, with a marked seasonality in the conformation of new interactions. Within the network, we differentiated four communities that included individuals of all the species. Regarding the transmission of pathogens, we observed the important role that fallow deer could be playing in the maintenance and transmission of pathogens to livestock. The present work shows the need to consider different types of methodologies in order to understand the complete functioning of the network of interactions at the wildlife/livestock interface. It also provides a methodological approach applicable to the management of shared diseases.

## 1. Introduction

The transmission rate of pathogens is dependent on two main parameters: the interaction rate between infectious and susceptible individuals and the probability of infection during each effective interaction [1]. The reliable estimate of these parameters has become a challenging task for epidemiologists, mainly when multiple and wild species are involved [2,3]. The transmission of a pathogen can occur directly (close contacts, within a few meters) and/or indirectly (transmission through the environment or through vectors), and sometimes by either method [4,5,6]. Therefore, the precise estimation of the transmission rate may require recording and quantifying of both direct and indirect interactions, which can then be used as a proxy of the risk of pathogen transmission [7]. Among the devices used to detect these interactions, the recently developed global positioning system (GPS) technology and proximity loggers can collect data with high spatio-temporal precision [8]. 

The information collected by GPS technology can be used to detect indirect interactions between individuals in a spatial scale, through the study of habitat selection and/or the home range overlap [9,10], or even in a spatio-temporal context, through the study of individuals co-location [11]. In the last decade, proximity loggers have appeared as a technology capable of detecting close interactions among hosts and their temporal dimension, providing relevant data for understanding the pathogen transmission, e.g., duration of the interaction [12]. However, this technology itself does not provide geospatial information of the interactions [13], so in order to obtain that data, it should be associated to GPS devices. Proximity loggers can also be used to detect indirect interactions between individuals and the environment through the monitoring of specific aggregation points in a spatio-temporal context, e.g., water points [14]. Overall, all these technologies are relevant to characterise the network of interactions within a community of individuals, since each of them is able to provide relevant and supportive information about host social structure [15] that is fundamental in determining how pathogens spread and persist and in parameterizing realistic epidemiological models [16]. 

The social network analysis (SNA) is a valuable approach that, in a broad sense, allows to describe the interactions among the individuals of a group (i.e., social structure), so the collective behaviour of the group can be studied [17,18,19]. In epidemiology, SNA is a useful analytical framework to characterize the network of interactions due to its ability to identify individuals and communities with a key role in pathogen transmission [18,20]. However, most of these applications of SNA in epidemiology have used static networks, that is, the assessment of the network structure and characteristics without the consideration of the time when the interactions have occurred. Nevertheless, the temporal changes in the interactions structure inevitably affect the dynamic of infection [21,22], by changing the properties of the network, that result in speeding up or slowing down the transmission rates [23]. Thus, to better determine how an infection spreads, moving from a static network to a dynamic one is required [24]. 

In Central and Southern Spain (hereafter CSS), farmed cattle in extensive conditions share space and resources with wild ungulates [25,26], namely red deer (*Cervus elaphus*), fallow deer (*Dama dama*) and wild boar (*Sus scrofa*) [27,28]. Wild reservoirs have been widely recognized as responsible for pathogen transmission and maintenance in this complex epidemiological scenario [29] and therefore, the correct management of diseases implies the comprehension of the process of transmission among species. Previous studies quantified the interaction among wild and domestic ungulates, showing a high overlap in home ranges and habitat selection, mainly in autumn [10,30], with the high relevance of water points for individual aggregation and subsequent frequency of interactions [11,30,31]. Characterising the network of interactions in such multi-host-environment system using dynamic SNA can contribute to obtaining a more realistic and precise context within which to study pathogen spread. Specifically, we aimed to use data obtained from GPS, proximity collars and proximity loggers located in wildlife and livestock aggregation points to describe (i) the specific role of each species in the conformation of the network, and (ii) the spatio-temporal dynamic of the network and its potential implications for the transmission of a theoretical pathogen for different probabilities of infection. 

## 2. Results

**Figure 1:**
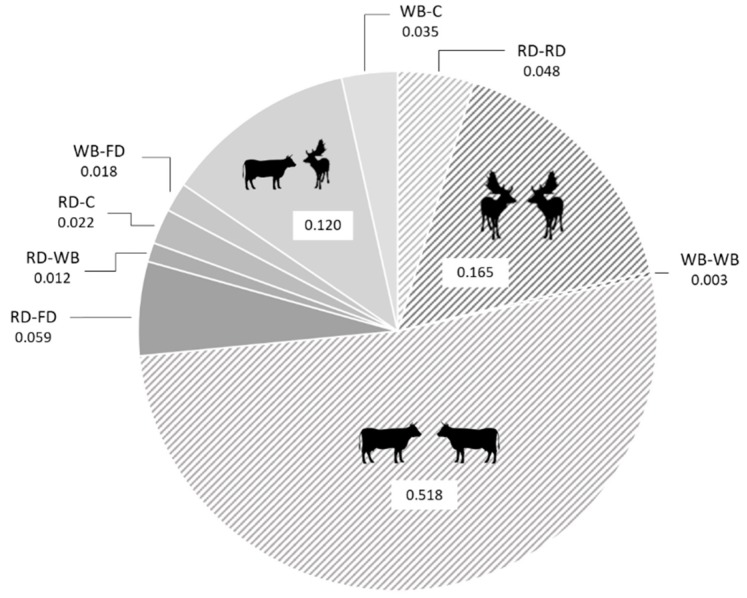
Frequency of appearance of different types of interactions (number of interactions per type divided by the total number of interactions recorded) according to the species involved (RD: red deer; FD: fallow deer; WB: wild boar; C: cattle). The striped background represents intraspecific intergroup interactions and the plain background represents interspecific interactions.

**Figure 2:**
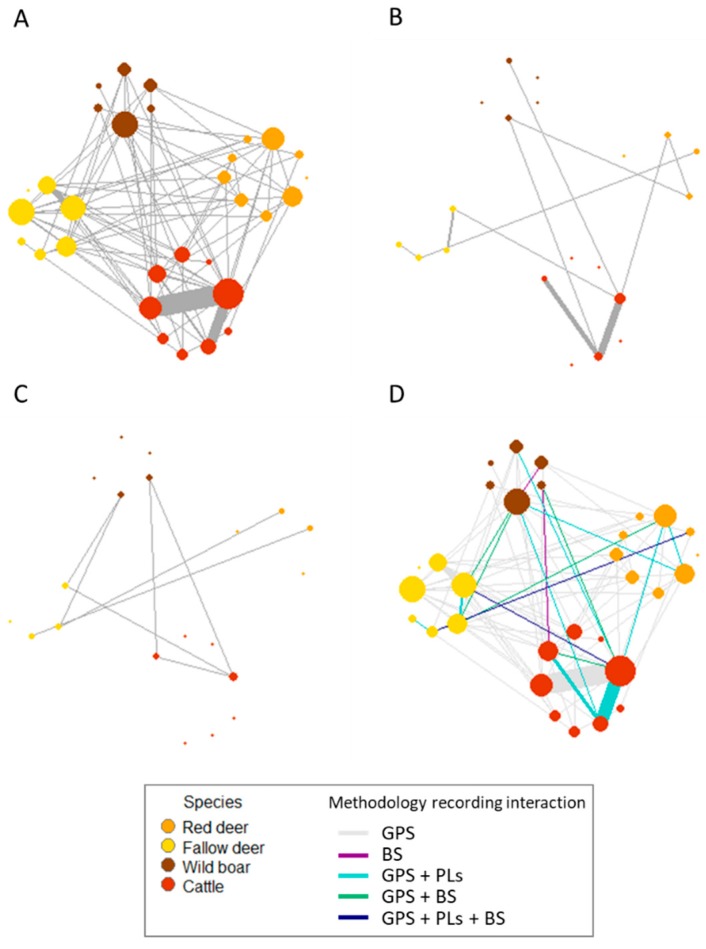
Social networks (grouped by species) of the interactions obtained throughout GPS technology (**A**), proximity logger collars (**B**), base stations (**C**) and the combination of the three methodologies (**D**). The width of the edge is proportional to the number of the interactions between two nodes. The size of the node is proportional to the degree. The colours of the nodes represent the different species. In the global network (**D**), the colour of the edge represents the methodology that record that interactions (GPS: Global Positioning System, PLs: proximity logger collars, BS: base stations).

Using the information from the GPS collars 1016 interactions were recorded. In the case of proximity collars (“PLs” hereafter) 169 interactions were registered. Finally, 17 interactions between individuals were recorded through proximity loggers set in environmental aggregation points (also known as base stations, “BS” hereafter). From the 31 individuals collared (with GPS or PLs collars), all of them interacted with at least one other collared individual, except one fallow deer and one red deer. The most probable interactions detected, were the intraspecific interactions among cattle and among fallow deer groups (see Figure 1 and Figure 2 (size of edges among nodes)). Although interspecific interactions were less frequent, the third most frequent interaction detected was among cattle and fallow deer (see Figure 1). 

Regarding the SNA representation, the global network had 29 nodes and 81 edges (Figure 2). Although the GPS technology made the bigger contribution in number of nodes and number of edges, this technology did not detect all the interacting pairs of nodes (see Figure 2D). The descriptive parameters of the network showed that the structure of the global network is sparse according to the low density (0.20), an intermediate clustering coefficient (0.52), and a large diameter (25) and mean path length (2.61). In addition, the network showed positive assortative values (0.13). 

**Figure 3:**
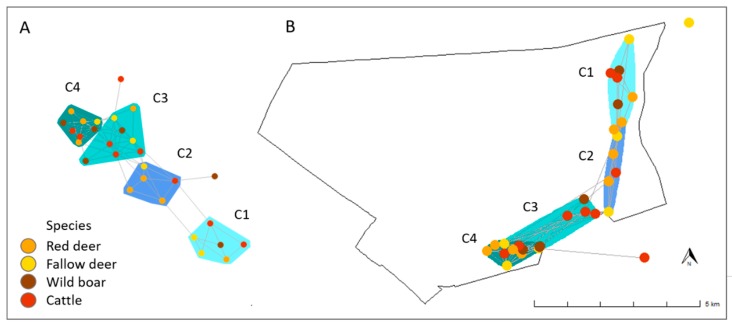
Characterization of the communities conformed by the targeted individuals (by species) in the study area (**A**), and geographical distribution of these communities (**B**). The colours of the nodes represent the species; communities are represented by different coloured shadows (**C1**, **C2**, **C3** and **C4**). The distribution of the nodes in the space in the image (**B**) corresponds to the geographical coordinates where animals were captured.

Statistical differences among species were observed for the eigenvector centrality value, with the cattle the species with the highest values (F_3,98_ = 7.57, *p* < 0.01) (see Supplementary Material: Figure S2). We did not observe any statistically significant difference among species in the rest of the centrality measures (degree: F_3,98_ = 1.23, *p* = 0.30, closeness: F_3,98_ = 0.18, *p* = 0.91, and betweenness centrality: F_3,98_ = 0.26, *p* = 0.85). Four communities covering the whole trapping area were identified in the global network (Figure 3). From the 29 animals included in the network, two individuals (a female cow and a female wild boar) did not belong to any community (see Figure 3A). All the communities included individuals of all the species of study, except one community which did not include wild boar (see community 2 [C2], Figure 3A). 

**Figure 4:**
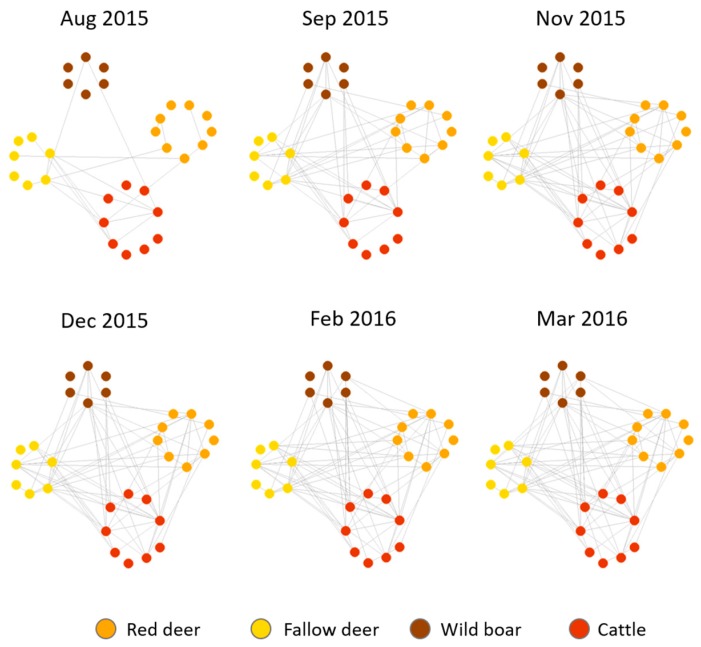
Dynamic network by month (Aug: August; Sep: September; Nov: November; Dec: December; Feb: February; Mar: March) of the interactions among targeted individuals. The colours of the nodes represent the different species of study. October and January are not represented since in those months, devices stopped sending information for battery saving purposes.

The dynamic network showed that edge formation and availability of individuals with which to interact was decreasing over time, presenting a seasonal pattern in the interaction rate (see Supplementary Material: Figure S1). We observed that most of the connections among nodes took place between August and November; however, new connections were established in winter as well (see Figure 4). It is also noticeable that interspecific interactions were frequent since the beginning of the study. 

### Simulation of Pathogen Transmission

**Figure 5:**
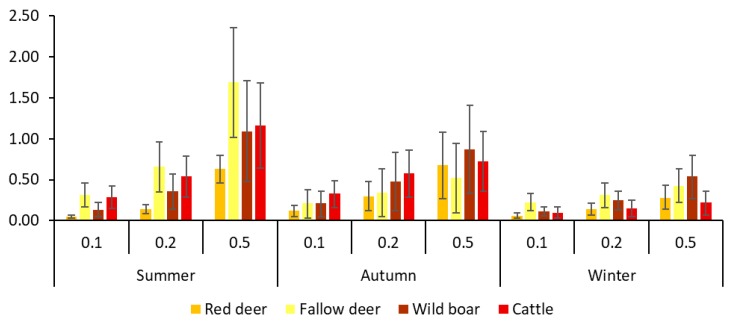
Mean number of infected cattle after the simulation of pathogen transmission by the different probabilities of infection and seasons, regarding the species initiating the infection.

The results of the simulation of pathogen spread showed differences in the number of infected cattle among seasons (F_2,271_ = 5.61, *p* < 0.01), after controlling for the probability of infection (F_2,271_ = 13.64, *p* ≤ 0.001) (see Figure 5 and Supplementary Material: Figure S3). Higher rates were observed in summer than in winter. When infection started in fallow deer, wild boar or cattle, a greater number of infected cows occurred in summer. However, an infected red deer could infect more cattle if this species was infected in autumn. A single infected fallow deer, wild boar or cow, could infect on average one cow in summer at the higher probability of infection modelled in our simulation (Figure 5). Finally, the wild boar and fallow deer were the species with the greatest probability of infecting cattle (Supplementary Material: Figure S3). The fallow deer infected a greater number of cattle during summer; however, wild boar took this role during the other two seasons, being the species that could infect more cattle during autumn and winter.

## 3. Discussion

We assessed a dynamic network of interactions among wild and domestic ungulates in an epidemiological complex scenario, for a better understanding of both the structure of interactions and of the underlying processes of pathogen transmission. The results allowed us to describe the role of the different species in the conformation of the network, its dynamics, and how this is reflected in a potential pathogen spread. Our approach enabled the description of the dynamic network of interactions using data of high spatio-temporal resolution, which is rarely found in the literature, quantifying and defining the connections among individuals and species. The complementarity of different monitoring technologies improved our ability to understand the transmission processes of pathogens among wildlife and livestock, helping to better target actions and control strategies at the wildlife/livestock interface. 

### 3.1. On the Study Approach

GPS and BS allowed us to estimate indirect interactions between collared individuals, whereas direct interactions were recorded through PLs collars. The definition of interaction was constrained by the spatio-temporal accuracy of the available technology, although we also used precise understanding of parameters of animal movement to determine the probability of interaction. However, from the epidemiological point of view, we cannot assume that the different types of interactions we evidenced weight the same in the network in terms of the real risk of pathogen transmission due to, for instance, differences in excretion ways among species [32], or the behaviour of the different species during the interaction (which could be recorded using camera traps [33,34]). Therefore, our simple simulation must be considered as a demonstrative exercise of the potential that our approach has to characterize networks of interactions for further risk modelling, rather than a real simulation of a model pathogen. Future research must be adapted to the specificity of the different pathogens including the different components of the systems (e.g., the environmental reservoir), precise transmission rates based on evidences and the specific type of interaction, asymmetry among species/individual in the transmission rates, specific behaviours during the interactions influencing the potential transmission of pathogens, individual variations in the risk of transmitting/becoming infected, etc. In summary, the definition of interaction for further risk modelling requires deep understanding of the nature of both the pathogen and the epidemiological system. 

### 3.2. On the Technologies

The implementation of efficient strategies to control pathogen transmission between wildlife and domestic animals requires the study and understanding of all the possible transmission routes for specific pathogens and systems. Nowadays, there are very useful technologies to study interactions at the wildlife/livestock interface with their specific strengths and weaknesses [15]. The combined use of these technologies is not common [13,14] but could be needed specially when studying pathogens transmitted both by direct and indirect pathways. In our case study, 63.4% of the interactions recorded with GPS devices took place in areas further than 150 m from a water point (data not shown). These would be not recorded if only water points were to be monitored, for instance only using BS [35]. It is likely that the omission of such information could result in different conclusions being made about the conformation of the network of interactions and therefore pathogen transmission. This problem could be overcome by combining the use of GPS, PLs and BS, or at least a methodology able to sample both direct (e.g., PLs) and indirect interactions (GPS) [13,36]. The present work combined these technologies in a multi-host scenario, in order to obtain a precise characterization of the dynamic network of interactions that is required to simulate pathogen spread. Due to the differences in the resolution of each methodology in our study, this work should be seen as a study of the complementarity use of methodologies, not as a comparison of them. 

### 3.3. Social Network Analysis 

A rapid interconnection among the monitored specific groups was detected during the first weeks of the study (Figure 4 and Supplementary Material: Figure S1). This suggests that in a few weeks an infected animal could introduce a pathogen to all species, particularly in summer and autumn. Previous studies also described the relevance of these seasons in explaining interspecific potential overlap, abundance and aggregation patterns in DNP [10,11,31,37]. 

We observed interactions among all the species, the most frequent interactions being the intraspecific of cattle and fallow deer and the third most frequent interaction among cattle and fallow deer. The high frequency of interspecific interactions among these two species could be a consequence of their gregarious tendency in open habitats [38,39], which can result in mixed groups. This result is very relevant from an epidemiological point of view since the high rate of interaction between groups and with cattle could confer to this species a crucial role in the maintenance of, for instance, TB that is the most relevant disease in ungulates in the study area [27], and other shared infections in the domestic compartment in DNP [40]. However, this quantitative approach may not consider differences in between-species rates of transmission. For instance, wild boar present high excretion rates of TB in DNP [41], and therefore risk of transmission per contact maybe be higher. 

Under our criteria to define interactions, we evidenced that indirect interactions were more frequent than direct ones, so the global network of interactions was constituted predominantly by indirect interactions. Previous studies suggested that direct interactions in the wildlife/livestock interface are not common in Mediterranean environments [25,30], neither in other European areas (for instance those involving European badgers *Meles meles* [34,36]). This is usually explained by the different daily activity patterns between wild and domestic species, and by avoiding behaviours among species. This circumstance reduces the likelihood of interspecific transmission, especially with pathogens that require direct interactions to be transmitted. However, the interactions registered between the collared individuals included in this study are only a representation of the real interactions taking place in the field, so the real epidemiological risk could be higher than presented here. 

The global network showed a sparse structure, suggesting that individuals tend to interact inside groups. This finding is common in social networks, especially with gregarious species like the ones studied here [42]. Four interconnected communities were identified in our network, all of them including individuals of all the targeted species (except community 2). The sparse structure observed is likely related with established communities of host (Figure 3), that are spatially structured around key resources. The distribution of essential resources describes the individuals’ distribution, abundance and aggregation of ungulates in DNP [31,37]. This is also supported from an epidemiological point of view, by the spatial pattern observed for the molecular types of *Mycobacterium tuberculosis* complex evidenced in DNP [28], which was observed to be relatively species-independent. The existence of “close” communities in the short term has epidemiological advantages since the potential spread of infections is expected to be slow and spatially limited [43]. However, in a territory with drastic environmental changes and human disturbances (like most of the Mediterranean area) the social structure of hosts can be seasonally altered, promoting interactions among individuals from different communities and enlarging the potential for pathogen transmission. Therefore, in the long term the probability of interaction between different host communities is relevant to the risk of pathogen transmission. For instance, long term studies are showing a significant spatial spread of the prevalence of TB from North to South in wild reservoirs in DNP [44]. This suggests that biosafety measures aimed to reduce the risk of interaction between wild and domestic animals, and therefore risk of transmission [45], should be implemented, since movement of individuals in response to environmental changes or human disturbance could promote the connection of previously isolated host communities in free ranging populations.

Regarding the role of the different species in the network, we did not observe statistical differences among species in the centrality measures of nodes: degree, closeness or betweenness. This means that in our demonstrative network all the studied species have a similar role in connecting different compartments [16]. However, we found statistical differences in the eigenvector centrality, with the cattle the species with higher values. Eigenvector centrality is a second-order measure of the connectivity of the individuals, which imply that cattle is connected to other highly relevant individuals in the network in terms of the number of interactions (as fallow deer; see Figure 1) [16]. This result shows how susceptible livestock could be to the entry of a pathogen in the network, especially if infection comes from fallow deer. 

### 3.4. Simulation of Pathogen Transmission

The exercise of simulating the transmission of a pathogen was useful to visualize the epidemiological consequences in the described network. We found differences in the average number of cattle being infected among seasons and among the species initiating the infection, evidencing the relevance of summer, and of fallow deer and wild boar for pathogen transmission to cattle. These results confirm the relevance of fallow deer and wild boar in the potential spread and maintenance of different pathogens in DNP. In the case of TB, the relevance of wild boar as a TB reservoir in DNP is justified by its high prevalence and diversity of shedding routes (e.g., [41]). However, in an Iberian context, red deer due to its higher density compared to wild boar and its capacity as super-shedder [32] may pose a significant risk for pathogen transmission, and therefore its potential role for transmission should be not considered negligible. Fallow deer still deserve more studies to disentangle its capability for transmission given the high potential suggested by our results, that in a Mediterranean context, could suppose a high epidemiological risk for cattle at a local scale.

The results reported here describing from a dynamic perspective the hosts’ population spatio-temporal structuration in DNP must be considered a first approach which requires further steps to simulate specific pathogens. We evidenced that the information collected by GPS technology and PLs can successfully be used to detect interactions between individuals at the spatio-temporal scale, providing relevant data for understanding pathogen transmission. This is considered a further step in the scientific based control of infections at the wildlife/livestock interface. 

## 4. Materials and Methods 

### 4.1. Study Area

**Figure 6:**
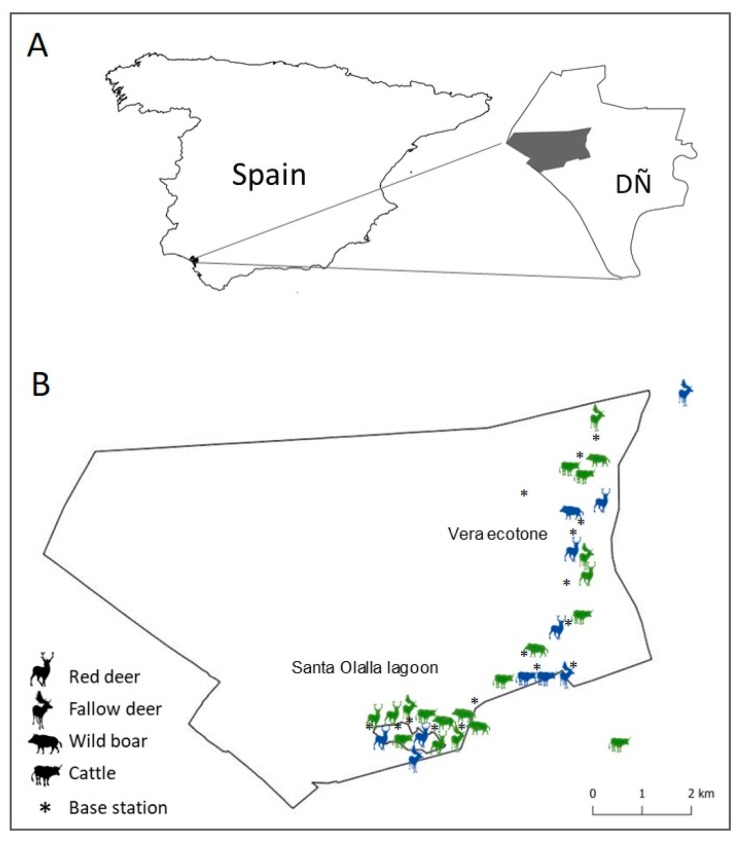
Study area location (**A**), and animal and base station locations in the study area according to species and type of device (**B**). In the image B locations of individuals correspond to the capture location, shapes correspond to the species, and colours correspond to the type of device (green: GPS-proximity-GSM collars; blue: GPS-GSM collars).

The study was performed in Doñana National Park (hereafter, DNP; 37° 08’ N; 6° 47’ W), which is located between Huelva and Sevilla provinces, south-west of the Iberian Peninsula (Spain) (see Figure 6). DNP has a total extension of 54,252 ha, with a wide variety of biotopes (marsh, beach, scrub and dunes), which maintains a great biodiversity of animals and plants. Within the group of ungulates, DNP maintains a moderate density of red deer (6.3 individuals/100 ha, coefficient of variation [CV] 23.54%), fallow deer (3.9 individuals/100 ha, 25.25% CV) and wild boar (5.7 individuals/100 ha, 20.63% CV) [46]. Also in DNP, husbandry of an endemic breed of cattle known as “vaca marismeña” is practiced. This domestic ungulate is kept in extensive conditions all year round, except for sanitary inspections that are carried out twice a year. The density of cattle in the study area is 2.26 individuals/100 ha [46]. 

DNP borders on its west part with the Atlantic Ocean, causing its climate to be considered dry sub-humid Mediterranean with Atlantic influence. The average annual temperature is 17 °C (4.6–32.6 °C), and the average annual precipitation is 549 mm with high intra and interannual fluctuation (252–1027 mm) (http://icts.ebd.csic.es/datos-meteorologicos). These irregular water inputs determine the dynamics of the marshland, therefore in the wet season (winter–spring) the marsh is flooded and the ungulates are aggregated in the scrub areas, but in the dry season (summer–autumn), only certain water points and meadows maintain water, which makes these areas attractive for multiple species [10].

### 4.2. Animal Capture and Monitoring

From August 2015 to July of 2017, we followed up 31 individuals of the four species of interest: 9 adult red deer (four males and five females), 7 adult fallow deer (three males and four females), 6 adult wild boar (three males and three females) and 9 cows (8 adult females and one young male). Animal captures were performed specifically in “la vera” ecotone (it is the transition pastureland that connects the scrubland and the marsh) and a nearby freshwater lagoon (Santa Olalla, see Figure 6B). In addition, 16 aggregation points for both wild and domestic ungulates, according to the risk resources for animal aggregation described by [37] and covering the home range of the targeted individuals, were monitored using proximity loggers (see Figure 6B). 

The animal captures were carried out by specialized scientists (B and C experimentation categories) according to the EC Directive 86/609/EEC for animal handling and experiments, following the protocol approved by the Animal Experiment Committee of Castilla-La Mancha University and by the Spanish Ethics Committee (PR-2015-03-08). Three methodologies were used to handle the animals: (i) red deer and fallow deer were stalked and then anesthetized with an anaesthetic rifle; (ii) the wild boar were captured by padded foothold cage traps previously baited with corn; and (iii) finally, cattle were collared during the annual management of routine veterinary inspections. Red deer and fallow deer were anesthetized with a combination of xylazine-ketamine following the protocol described by [47] and wild boar with tiletamine-zolazepam and metomidine following the protocol described in [48].

Three types of monitoring devices were used in this study: (i) GPS-GSM collars (five red deer, three fallow deer, one wild boar, two cows), (ii) GPS-proximity-GSM collars (previously described as “PLs”, four red deer, four fallow deer, five wild boar, 7 cows) and (iii) base stations (BS). All of them were developed by Microsensory S.L. (Microsensory System, Spain). Finally, from the 16 BS set in aggregation points, 11 were placed in water points, two in food points and three in points with a shared used of water and food. 

GPS collars were set up to record one location every two hours. For each location, collars also recorded the ID of the individuals, date, time (solar time), geographical coordinates and the location acquisition time. When this parameter was >254 s, the location data was removed since it was considered to be imprecise. Location data of the day in which devices were deployed were also removed in order to avoid possible biases caused by anomalous animal behaviours after handling. In order to save battery power and extend the period of monitoring, all devices were configured to stop sending information during one out of every three months (two months working, one month resting; see Supplementary Material: Figure S1). The mean positioning error of these devices was 26 m according to field trials carried out at the beginning of the captures following the protocol presented by [10]. 

Proximity logger collars were set up according to two parameters: the time receiving window (the period in which PLs were able to detect other individuals wearing another PLs within the detection range) and the frequency of UHF beeps emission. For the first parameter we used 10 seconds per minute, and for the second parameter PLs sent one beep every minute. Every time an individual’s PLs detected the beep of another individual’s PLs, the PLs of the first individual recorded the ID of the second, the date and time of the encounter and the RSSI (received signal strength indicator). The RSSI can be used as a probabilistic measure of the distance of the encounter by a previous calibration between this parameter and the distance between devices in specific habitats [8]. Similarly as with the GPS collars, PLs were configured to rest one month each season; which enabled the monitoring of individual animals over a longer period (i.e., including the four seasons, approximately one year). In the case of BS, only the time receiving window was configured, since these devices only registered other devices nearby, but do not transmit a proximity signal. 

### 4.3. Definition of Interactions

GPS and BS allowed us to estimate indirect interactions between collared individuals, whereas direct interactions were recorded using the PLs collars.

#### 4.3.1. GPS Technology

To record indirect interactions between individuals using GPS information, the first step is to define the spatio-temporal window between consecutive relocations to be considered as an interaction. The spatial window considered was 52 m according to the mean positioning error of the devices (26 m radius). The temporal window was established as two hours due to the frequency of location fixation. Briefly, for each relocation of a reference individual, we looked for a relocation from a different individual inside the spatio-temporal window established. For each interaction, we noted the ID of the interacting individuals, the Euclidean distance between locations, date, time and the coordinates (for further details, see [11]). The analyses were performed with R software 3.5.3 [49].

#### 4.3.2. Proximity Technology

Direct interactions were recorded using PLs. To describe direct interactions, we need to define the distance between individuals detected by PLs that may lead to close interactions. The distance between individuals was calculated throughout the RSSI parameter following the methodology previously published in [8]. In the present study, the distance between individuals to be considered as interacting animals was 20 m. For that, we used as reference the information presented recently by [50] who established that wild boar walk in DNP on average 10 m per minute; therefore, two interacting animals would normally not be farther away than 20 m in a minute (temporal resolution of PLs) if they are walking in opposite directions after having interacted. 

For BS, we defined indirect interactions as two animals using the same resource (at a pre-established distance) within a temporal window. For BS, the spatio-temporal window was defined as 20 m and two hours, according to previous specifications in PLs and GPS-recorded interactions, respectively.

### 4.4. Network Characterisation and Data Analysis

We first quantified the different types of interactions according to the species involved. Then, using the “igraph” R package [51] we constructed a static network combining the information from all of the methodologies (GPS, PLs and BS) (global network, hereafter) and an independent one for each methodology in order to visualize the contribution of each methodology to the global network. For all the networks we estimated the metrics of the nodes (degree, closeness, betweenness centrality and eigenvector centrality) and the metrics of the network (diameter, density, clustering coefficient, the average path length and the degree assortativity) (for more information about metrics, see Table 1). Statistical differences in node metrics among different host species were checked using linear models in R software [49]. 

In the graphical visualisation of the networks, nodes represent individuals (by species) and edges represent an interaction recorded between two nodes. In the global network, we represented edges according to the methodology that recorded the interactions. In all the networks, the size of the nodes represents the degree and the weight of the edges represents the number of interactions between nodes. Additionally, by means of the global network we identified the communities (i.e., groups of individuals of the same or different species that are more closely connected and therefore, with more potential to share pathogens) throughout the “Walktrap” algorithm in igraph. This function detects subgroups inside the network through the idea that shorter random walks between nodes will occur in the same community [51]. 

Finally, in order to study the dynamics of the interactions, the formation of new edges in the network over time and the implications of such evolution for the potential transmission of pathogens, using all interactions and the week as temporary unit, we applied the function “tEdgeFormation” of the tsna R package [55] on a dynamic network created with the R package networkDynamic [56]. 

### 4.5. Simulation of Pathogen Transmission

One of the most useful applications of dynamic networks in epidemiology is the possibility to simulate and explore the transmission of pathogens on the basis of the different parameters that characterise them like, the probability of transmission, the first individual/s infected or transmission within a specific period of time [24]. In the dynamic network we simulated the transmission of a theoretical pathogen transmitted through both direct and indirect pathways, according to our spatio-temporal definition of direct and indirect interaction, to explore the potential role of wild species in the transmission to cattle. We also included the cattle as a possible donor in order to control the intraspecific transmission. The simulation was performed following the approach provided by [57]. Three scenarios of theoretical probability of infection were considered: 0.1, 0.2 and 0.5. The probability of infection given interaction in wild conditions is a difficult parameter to estimate, so we selected these values according to a range of β parameter presented in previous works, which modeled the transmission of a pathogen transmitted through direct pathways (foot and mouth disease [FMD]; [58,59]) and indirect pathways (animal tuberculosis [TB]; [60]). These are two relevant infectious diseases in Spain since, although this country has been FMD free since 1986, there is a real risk of pathogen introduction through animal imports [61]. Regarding TB, wild ungulates have been described as having the ability to maintain this disease in the absence of cattle. This partly explains the inefficacy of the national eradication program implemented on cattle [27]. Using this range of β parameter we cover the potential transmission of pathogens transmitted through direct or indirect interactions. We simulated 100 times the spread of the pathogen using all the nodes of the global network as the initial infected individual. After the simulation we calculated the average number of cattle being infected under the different probabilities of infection, according to the species that initiated the transmission. The simulation was performed for three seasons conditions: summer, autumn and winter. We did not include the simulation for spring due to limitations in the number of monitoring devices (GPS collars and PLs) properly working in the field from March 2016 (see Supplementary Material: Figure S1). Finally, using a linear model in R we checked for statistical differences in the number of cattle infected according to the species starting the infection and season, considering all the probabilities of infection in all the species, and controlling for this factor in the model. 

## Figures and Tables

**Table 1:** Glossary of node and network metric terms.

Term		Range	Definition *
Node properties	Degree	0 to ∞	Number of contacts of a specific node.
	Closeness	0 to 1	The ratio of how many nodes are connected directly with a reference node.
	Betweenness centrality	0 to ∞	Number of shortest paths between the nodes of the network that pass through a specific node.
	Eigenvector centrality	0 to 1	A measure of how connected a specific node is to other nodes highly relevant in the network.
Network properties	Diameter	0 to ∞	The shortest path length inside the network.
	Density	0 to 1	Number of contacts in the network regarding the total number of contacts that could had happen.
	Clustering coefficient	0 to 1	Sum of the proportion of nodes that are connected to another node.
	Average path length	0 to ∞	Average number of contacts that take place through the shortest path among all the nodes in the network.
	Degree assortativity	−1 to 1	Tendency of a specific node to contact with nodes which have similar degree values to the reference node.

* Based on the descriptions provided in [18,52,53,54].

## Supplementary Materials

The following are available online at https://www.mdpi.com/2076-0817/9/2/120/s1, Figure S1: Node availability (A) and edge formation (B) over time (week as temporal unit) for the global network, Figure S2: Node metrics values by species, Figure S3: Average number of infected cattle predicted by the simulation procedure according to season (A), the first species infected (B) and the theoretical probability of infection (C).
