# Supplementary material for: Dynamic Network of Interactions in the Wildlife-Livestock Interface in Mediterranean Spain: An Epidemiological Point of View
Source: Pathogens. 2020 Feb 13;9(2):120. doi: 10.3390/pathogens9020120 (PMC7169396; doi:10.3390/pathogens9020120)
Supplement: Supplementary file 1 [file pathogens-09-00120-s001.pdf]

sustainability

MDPI

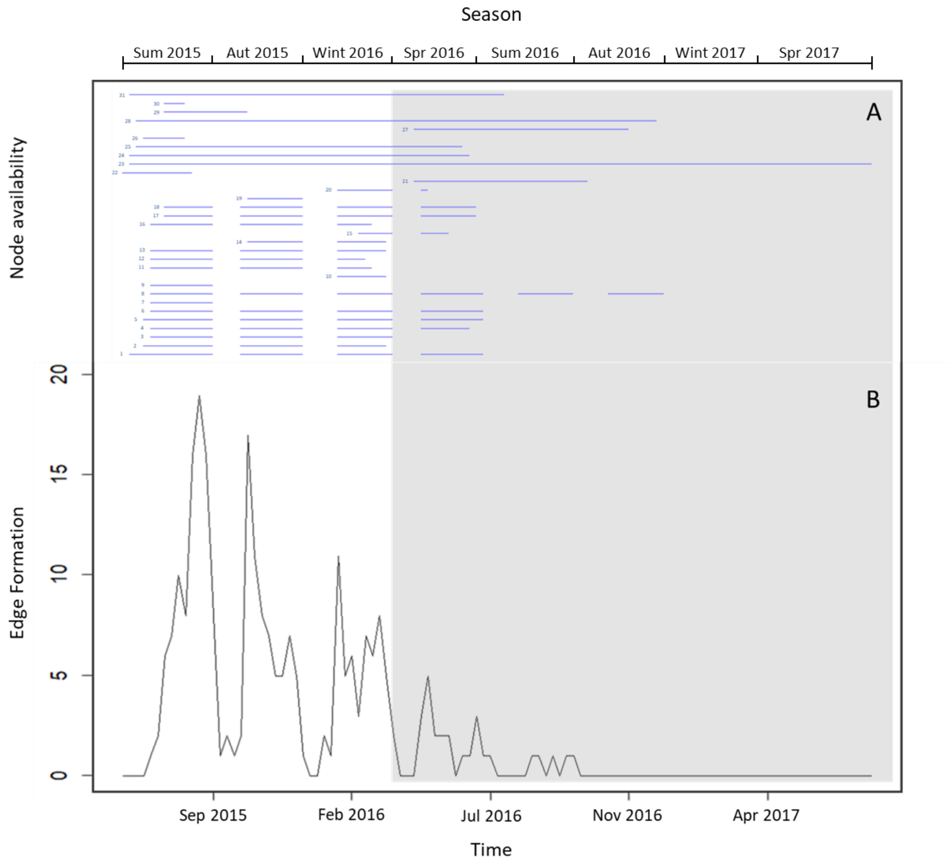

Figure S1. Node availability (**A**) and edge formation (**B**) over time (week as temporal unit) for the global network. The numbers in A graph correspond to the ID of the individuals. The discontinuous availability in graph A corresponds to the months in which devices were off for battery saving purposes. Seasons for the period of study are represented over the graph (Summer: Sum; Autumn: Aut; Winter: Wint; Spring: Spr). Grey shadow marks the period in which the number of available devices did not allow for further analyses.

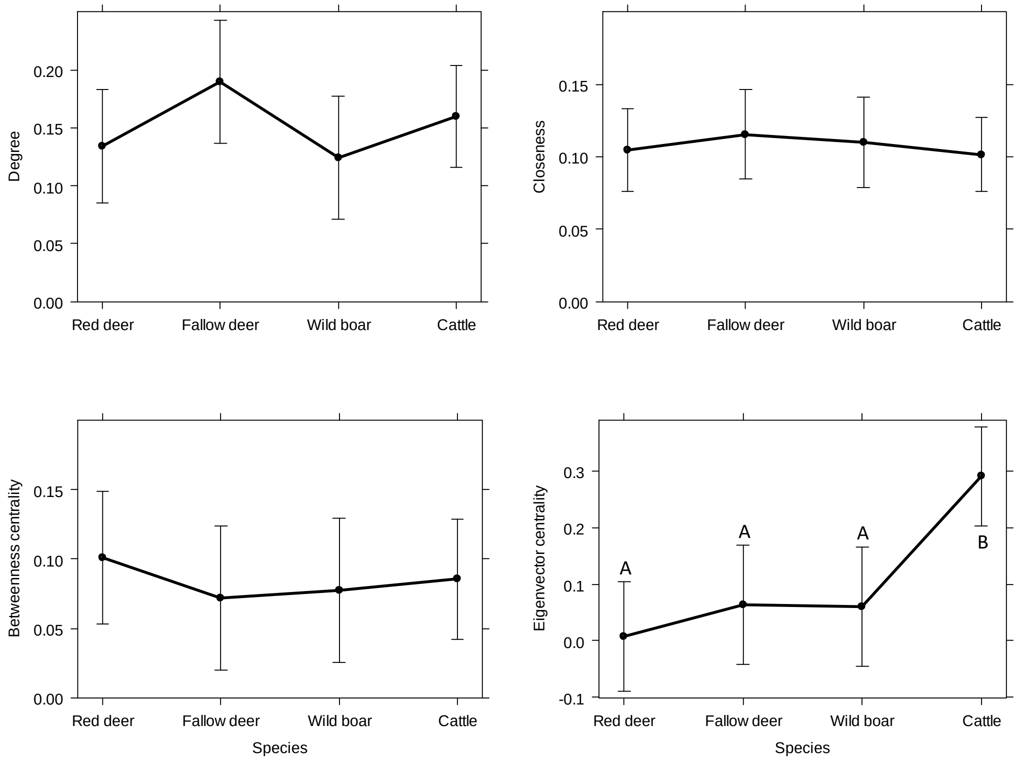

Figure S2. Node metrics values by species. The graph represents the predicted values and standard error (95% CI) according to linear models. Different letters show significant differences among categories ($p < 0.05$); no letters indicate no statistical differences among categories ($p > 0.05$) in all pairwise comparisons.

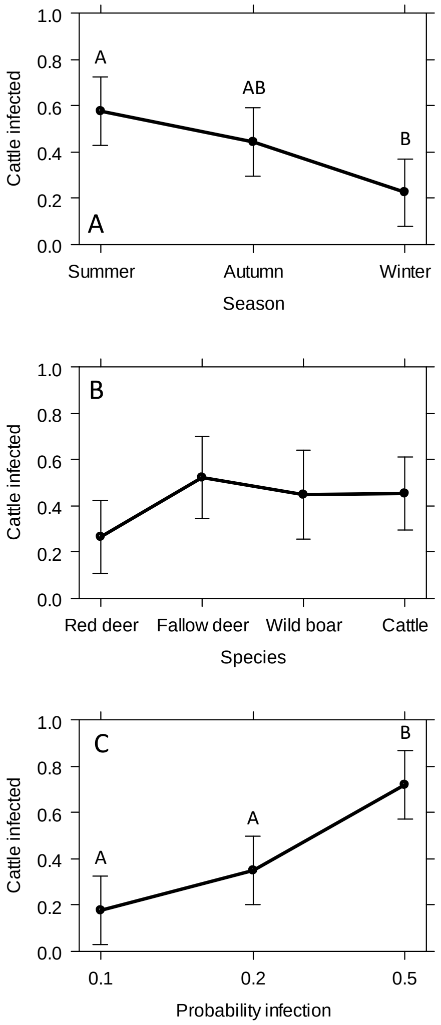

Figure S3. Average number of infected cattle predicted by the simulation procedure according to season (**A**), the first species infected (**B**) and the theoretical probability of infection (**C**). The graphs represent the predicted values and standard error (95% CI) according to linear models. Different letters show significant differences among categories ($p < 0.05$); no letters indicate no statistical differences among categories ($p > 0.05$) in all pairwise comparisons.
